# A gene expression restriction network mediated by sense and antisense Alu sequences located on protein-coding messenger RNAs

**DOI:** 10.1186/1471-2164-14-325

**Published:** 2013-05-11

**Authors:** Kung-Hao Liang, Chau-Ting Yeh

**Affiliations:** 1 Liver Research Center, Chang Gung Memorial Hospital, Taipei, Taiwan; 2 Molecular Medicine Research Center, Chang Gung University School of Medicine, 199 Tung Hwa North Road, Taipei 10507, Taiwan

**Keywords:** Antisense Alu, Gene expression restriction, Double-stranded Alu, Alu-carrying protein-coding RNA

## Abstract

**Background:**

Alus are primate-specific retrotransposons which account for 10.6% of the human genome. A large number of protein-coding mRNAs are encoded with sense or antisense Alus in the un-translated regions.

**Results:**

We postulated that mRNAs carrying Alus in the two opposite directions can generate double stranded RNAs, capable of regulating the levels of other Alu-carrying mRNAs post-transcriptionally. A gene expression profiling assay showed that the levels of antisense and sense Alus-carrying mRNAs were suppressed in a reversible manner by over-expression of exogenous sense and antisense Alus derived from mRNAs (Family-wise error rate P= 0.0483 and P < 0.0001 respectively). Screening through human mRNAs on the NCBI-RefSeq database, it was found that sense and antisense Alu-carrying transcripts were enriched in distinct cellular functions. Antisense Alu-carrying genes were particularly enriched in neurological and developmental processes, while sense Alu-carrying genes were enriched in immunological functions.

**Conclusions:**

Taken together, we proposed a novel Alu-mediated regulation network capable of stabilizing Alu-carrying mRNA levels in different cell types and restricting the activated expression levels of protein-coding, Alu-carrying mRNAs.

## Background

An intriguing characteristic of the human genome is its containing of vast numbers of Alus, a class of short-interspersed repetitive sequences with a length of 280~300 nucleotide bases [1-3]. More than one million copies of Alus altogether contribute 10.6% of the human genome [1,2]. Alus were retrotransposons evolved from a duplication of the 7SL RNA gene more than 65 million years ago [1-4]. The retrotransposition process of Alus relies on the machinery carried by the long interspersed nucleotide element 1 (L1), another retrotransposon which contributes 17% of the human genome [4,5]. Alus have diverse sequence variations [6,7]. A total of 213 Alu subfamilies have been reported based on a thorough computation of sequence homology in the human genome [2].

Alus were found in both genic and intergenic regions of the human genome [3], with a higher frequency in the former [8]. Intergenic Alus can be transcribed by polymerase III, yet the transposition activities have remained dormant [9]. Polymerase III-derived Alu transcripts are constantly shattered by *Dicer1* in normal human physiology, failure of which may result in Alu toxicity which in turn triggers geographic atrophy [10], an advanced form of age-related macular degeneration.

Genic Alus have been found in upstream and intronic regions [11], as well as exonic regions such as 5′ untranslated regions (UTRs) [12] and 3′UTR of messenger RNAs [3]. Alus in mRNAs are classified as exonic or exonized Alus, depending on whether they are embedded within a longer exon or are spliced into mRNAs as an individual exon. Exonized Alus have been shown to express only occasionally and have low copies of transcripts within cells [12,13]. Alus have been shown to encompass a 6-base sequence tag complimentary to one common seed of 30 human miRNAs [14]. Recently, long non-coding RNAs have been shown to be capable of binding to Alu-carrying mRNAs, thereby triggering the STAU1-mediated mRNA decay [15]. An analysis of human chromosomes 21 and 22 showed that genic Alus are particularly enriched in genes of metabolism, transport and signaling processes [16]. Despite these analyses, the cellular roles of genic Alus remain largely elusive [3,8,11]. Alus were once thought of as parasite-like, selfishly-replicated junk DNAs without prominent constructive roles to human cells [13,17].

Human mRNAs may carry Alus in either sense or antisense directions. In light of the thermodynamics properties of nucleotide base pairing, we were intrigued to ask whether these mRNAs form double stranded duplex longer than 290 bases, and if that happens, what their corresponding cellular roles could be? Despite the binding of two protein-coding Alu-carrying mRNAs had never been discussed previously to our knowledge, we conjectured that the resulting double stranded RNAs could trigger the post-transcriptional regulation of a large collection of protein-coding mRNAs carrying sense or antisense Alu elements, by offering potent sources of either *Dicer1*-created short interfering RNA (siRNA) [18-20], or *STAU1*-mediated mRNA decay [15]. Both mechanisms were originally proposed to address the binding of a non-coding and a protein-coding RNA.

An Alu-carrying mRNA may form a binding with multiple antisense Alu-carrying mRNA, and vice versa. Consequently, mRNAs with sense and antisense Alu elements produce a many-to-many network, where those with the sense elements are prevailingly regulated by those with the antisense elements, resulting in coordinated reaction. Such coordination has been postulated recently on the topic of micro RNAs (miRNA) against genes, pseudogenes and long non-coding RNAs which share the same miRNA targets [21,22]. Intriguingly, Vidal and colleagues showed that mouse and rat mRNAs carrying sense B1 repeats are expressed coordinately, reaching a maximum level in the G2 phase of the cell cycle [23]. Data showed that the B1 repeats are necessary rather than sufficient criteria for the coordination. It is worth noting that B1 repeats (~140 bases) were also originated from 7SL RNA gene [6].

## Results

### Strong sense-antisense bindings of Alu-carrying mRNAs predicted by RNA co-folding computation

**Figure 1:**
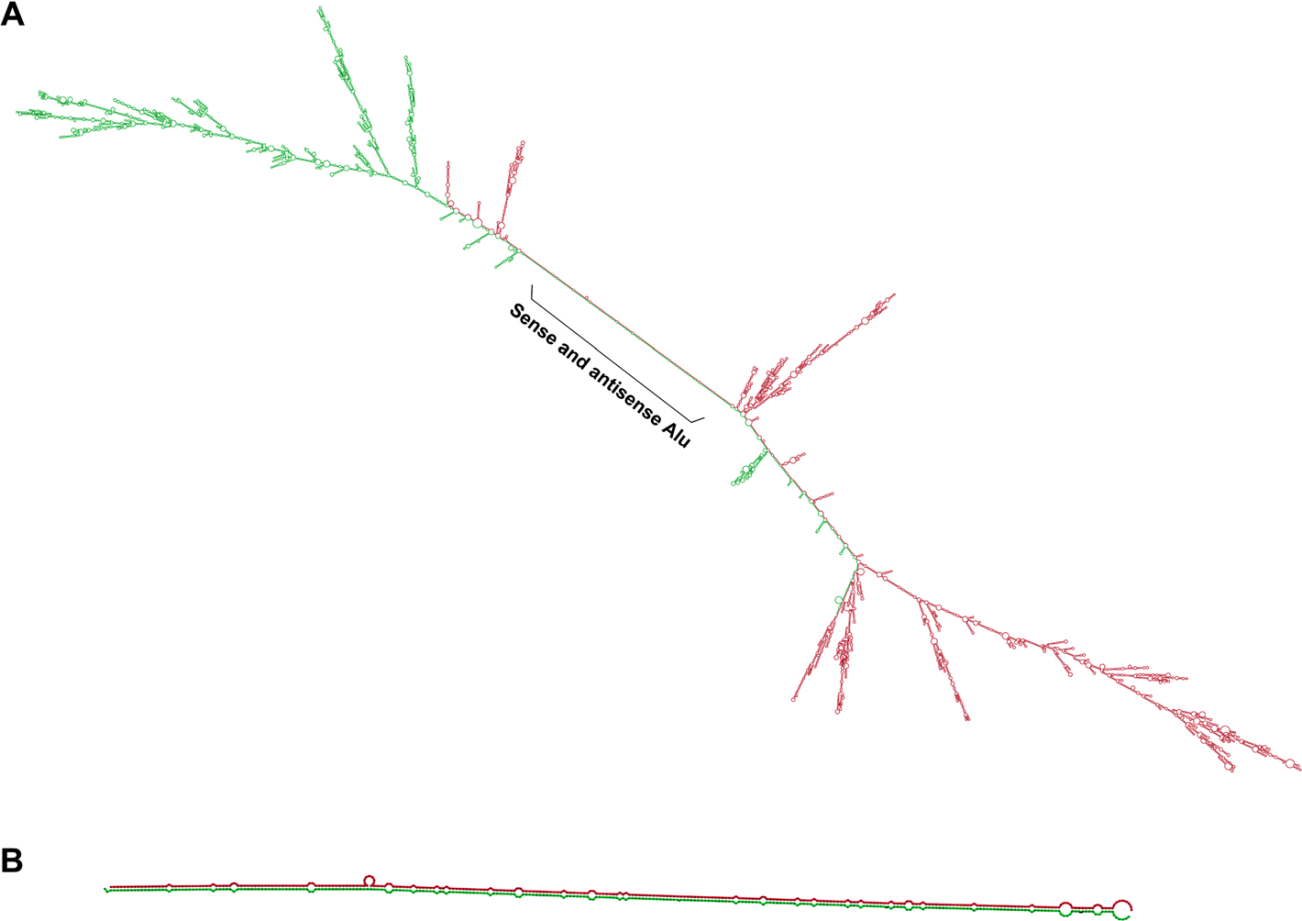
**Secondary structures of *PER2 *and *PCM1 *mRNAs predicted by a co-folding algorithm. (A)** The co-folding of full length mRNAs of *PER2* (Green) and *PCM1* (Red). A long line of duplex structure was observed. **(B)** A focused view of the duplex caused by the antisense base pairing of Alus on *PER2* (Green) and *PCM1* (Red).

The first conjecture was the binding of messenger RNAs carrying sense and antisense Alus. Inspired by Vidal and colleagues’ work on cell cycles [23], our exploration started from two genes carrying respectively the sense and antisense Alu elements, *PCM1* (which is known for its role on cell cycles) and *PER2* (a major gene in circadian cycles) (Table 1). The co-folding structure of the two full-length mRNAs was computed, showing a long formation of RNA duplex of 318 bases which clearly stood out from other local structures (Figure 1A). This duplex was formed by the base pairing of sense and antisense Alus (Figure 1B). The estimated free energy of the duplex is -461.3 kcal/mol. Deducting the free energy of the two elements in isolation (-102.2 and -123.4 kcal/mol respectively), the net change of energy (denoted as ΔG) is -235.7 kcal/mol [24] which indicated a strong encouragement of binding and provided positive evidence supporting the first conjecture.

**Table 1:** Two genes with Alu sense or antisense elements in the 3′UTR

**Gene symbol**	**Alu direction**	**RefSeq accession**	**ALU region**	**Length**	**3′UTR**	**Exon containing the ALU**	**GC%**
*PER2*	antisense	NM_022817.2	5318-5631	314	4005-6342	3856-6342	52.9
*PCM1*	sense	NM_006197.3	7691-8008	318	6497-8788	6472-8783	52.8

### Protein-coding mRNAs with Alu elements in opposite directions also carry distinct biological functions

The second conjecture was that the duplex of Alu-carrying mRNAs may trigger subsequent degradations of other Alu-carrying RNAs. If such mechanism exists, it follows that sense Alu-carrying mRNAs (referred to as Sens-alus) and antisense Alu-carrying mRNAs (Ant-alus) cannot concurrently stay in high concentrations in human cells. Instead, there are three possibilities: (i) Ant-alu high and Sens-alu low; (ii) Ant-alu low and Sens-alu high; (iii) both Sens-alu and Ant-alu are low. In other words, states (i) and (ii) represent the dominant expression patterns of only one Alu-carrying RNA species. As such, a RNA species might be enriched in certain pathways, while depleted in other pathways, resulting in different functional annotations of the two species. The corresponding null hypothesis is that states (i) and (ii) does not exist and their constituent genes are randomly scattered in a wide spectrum of biological categories and pathways. This hypothesis can be assessed by checking the over and under representation of genes in pathways and biological processes.

We screened Sens-alus and Ant-alus from the entire NCBI-RefSeq human mRNAs [25] using sequence homology search. A majority of these Alu elements reside in the 3′UTR (99%) and only 1% of them reside in the 5′UTR region. None of them were found to reside completely in the coding region. 689 Ant-alus and 771 Sens-alus were identified respectively, resulting in a total sum of 1460 genes which corresponds to 7.3% of human protein-coding genes (Additional file 1: Table S1, Additional file 1: Table S2). Computational analysis on a random selection of pairs of Ant-alus and Sens-alus showed that all of them can form computational predicted bindings with ΔG lower than -200 kcal/mol. In addition, 190 genes were found to have Alu elements in both sense and antisense directions (Additional file 1: Table S3).

Functional annotations of Ant-alus and Sens-alus showed that the two RNA species were differently distributed in multiple pathways and biological functions. Ant-alus were over-represented in multiple signaling pathways of neurotransmitters such as serotonine, gamma aminobutyric acid (GABA), glutamate, acetylcholine and cannabinoid. They were also over-represented in synaptic vesicle trafficking, opioid and (dopamine producing) pyridoxal phosphate pathways (P<0.05; Table 2). Ant-alus were under-represented only in the Huntington disease pathway (Table 2). Additionally, Ant-alus were over-represented in the biological processes such as dorsal-ventral axis, exocytosis, neurotransmitter secretion, organelle organization and vesicle mediated transport (Table 3). Ant-alus were under-represented in the biological processes of anion transport, nerve-nerve synaptic transmission and response to stimulus and toxins (Table 3).

**Table 2:** List of all pathways where Sens-alus and Ant-alus are enriched or depleted

**Pathway**	**Ant-alu P**	**Sens-alu P**
**Ant-alu enriched**		
p53 pathway	**0.0005**†	0.2470
Opioid prodynorphin pathway	**0.0015**†	0.6090
Muscarinic acetylcholine receptor 2 and 4 signaling pathway	**0.0018**†	0.2270
Pyridoxal phosphate salvage pathway	**0.0023**†	0.9260
Vitamin B6 metabolism	**0.0050**†	0.8910
Metabotropic glutamate receptor group II pathway	**0.0070**†	0.5520
5HT1 type receptor mediated signaling pathway	**0.0077**†	0.2930
Opioid proopiomelanocortin pathway	**0.0088**†	0.4040
Opioid proenkephalin pathway	**0.0088**†	0.4040
Endogenous_cannabinoid_signaling	**0.0132**†	0.3670
GABA-B_receptor_II_signaling	**0.0133**†	0.4560
Metabotropic glutamate receptor group III pathway	**0.0144**†	0.5340
Muscarinic acetylcholine receptor 1 and 3 signaling pathway	**0.0189**†	0.3270
Beta3 adrenergic receptor signaling pathway	**0.0210**†	0.3220
Cortocotropin releasing factor receptor signaling pathway	**0.0312**	0.3770
5HT4 type receptor mediated signaling pathway	**0.0341**	0.1540
Synaptic_vesicle_trafficking	**0.0372**	0.5960
p53 pathway feedback loops 2	**0.0407**	0.1580
Angiotensin II-stimulated signaling through G proteins and beta-arrestin	**0.0438**	0.5690
**Ant-alu depleted**		
Huntington disease	**0.0230**	0.3950
**Sens-alu enriched**		
Toll receptor signaling pathway	0.6120	**0.0042**
DPP_signaling_pathway	0.5960	**0.0210**
Nicotinic acetylcholine receptor signaling pathway	0.2970	**0.0225**
Gamma-aminobutyric acid synthesis	0.8130	**0.0229**
SCW_signaling_pathway	0.4430	**0.0289**
BMP_signaling_pathway-drosophila	0.4430	**0.0289**
Interleukin signaling pathway	0.5740	**0.0365**
Endothelin signaling pathway	0.3140	**0.0420**
DPP-SCW_signaling_pathway	0.5150	**0.0487**

†The accompanying False Discover Rate < 25%.

The P values were nominal values without being corrected for multiple comparisons.

**Table 3:** List of all biological processes where Ant-alus and Sens-alus are either enriched or depleted

**Biological process**	**Ant-alu P**	**Sens-alus P**
**Ant-alus enriched**		
exocytosis	**0.0003**†	0.0807
synaptic vesicle exocytosis	**0.0007**†	0.0333
neurotransmitter secretion	**0.0011**†	0.1060
polysaccharide metabolic process	**0.0022**	0.4470
organelle organization	**0.0033**	0.3170
establishment or maintenance of chromatin architecture	**0.0056**	0.3100
mammary gland development	**0.0090**	0.0841
glycogen metabolic process	**0.0153**	0.5300
protein targeting	**0.0183**	0.4670
dorsal/ventral axis specification	**0.0263**	0.2970
vitamin catabolic process	**0.0339**	0.9620
catabolic process	**0.0339**	0.9620
cellular amino acid catabolic process	**0.0385**	0.5430
**Ant-alus depleted**		
anion transport	**0.0069**	0.4800
visual perception	**0.0132**	0.5310
developmental process	**0.0133**	0.1630
nerve-nerve synaptic transmission	**0.0148**	0.3080
neuromuscular synaptic transmission	**0.0153**	0.5010
sensory perception	**0.0218**	0.4540
response to toxin	**0.0327**	0.5300
response to stimulus	**0.0351**	0.2500
cell motion	**0.0439**	0.2520
cell-cell adhesion	**0.0469**	0.3020
**Sens-alus enriched**		
cyclic nucleotide metabolic process	0.5180	**0.0040**
termination of RNA polymerase II transcription	0.5570	**0.0289**
response to interferon-gamma	0.3500	**0.0335**
meiosis	0.2210	**0.0346**
cytokine-mediated signaling pathway	0.3820	**0.0396**
RNA localization	0.2320	**0.0397**
**Sens-alus depleted**		
ectoderm development	0.1360	**0.0234**
mRNA 3′-end processing	0.2320	**0.0255**
mRNA polyadenylation	0.2200	**0.0275**
synaptic transmission	0.1390	**0.0285**
macrophage activation	0.4460	**0.0387**
cellular glucose homeostasis	0.0732	**0.0390**
embryonic development	0.4190	**0.0450**
defense response to bacterium	0.0749	**0.0457**

†The accompanying False Discover Rate < 25%.

The P values were nominal values without being corrected for multiple comparisons.

On the other hand, Sens-alus were over-represented in immunological pathways such as Toll-like receptors, Interleukin and endothelin signaling (P<0.05, Table 2). Sens-alus were not under-represented in any pathways. Sens-alus were also over-represented in biological processes related to cytokine-mediated signaling pathway, responses to interferon gamma and meiosis. Sens-alus were under-represented in synaptic transmission and ectoderm development (Table 3). Interestingly, Ant-alus were over-represented (P = 0.0007) while Sens-alus were under-represented (P = 0.0333) in the biological process of synaptic vesicle exocytosis (Table 3).

A scrutiny of the constituent genes revealed that the Sens-alus species has greater numbers of immune-related genes, particularly the Toll-like receptors, Cytokines and Cluster of differentiations, than Ant-alus (Table 4). On the contrary, the Ant-alu species has more embryonic stem cell-related genes than Sens-alus (Table 5). The distinct functional annotations of Sens-alus and Ant-alus in our analysis suggested that the insertion and maintenance of Alus in mRNAs in the two directions were not entirely random. Instead, these protein-coding genes might mediate regulatory processes via Alu elements for special functions.

**Table 4:** Comparison of immune-related genes in Ant-alus and Sens-alus

	**Ant-alus**		**Sens-alus**	
	**Gene symbol**	**RefSeq accession**	** Gene symbol**	**RefSeq accession**
V(D)J recombination			*DCLRE1C*	NM_001033855.1
Toll like receptors			*TLR6*	NM_006068.3
			*TLR7*	NM_016562.3
			*TLR10*	NM_030956.2
Interferon related			*IRF1*	NM_002198.2
			*IFIT3*	NM_001031683.2; NM_001549.4
Cytokines	*IL11*	NM_000641.2	*IL1R1*	NM_000877.2
	*IL28RA*	NM_170743.2;	*IL2RA*	NM_000417.2
		NM_173064.1;		
		NM_173065.1		
	*IFNAR2*	NM_207585.1	*IL6R*	NM_000565.2 ; NM_181359.1
			*IL10*	NM_000628.3
			*IL10RB*	NM_000628.3
			*IL13RA1*	NM_001560.2
			*IL17RA*	NM_014339.4
			*IL18*	NM_001562.2
			*IL23R*	NM_144701.2
			*CSF2RA*	NM_006140.4;
				NM_172245.2;
				NM_172246.2;
				NM_172247.2;
				NM_172249.2;
				NM_001161529.1;
				NM_001161530.1;
				NM_001161532.1;
Chemokines	*CCL22*	NM_002990.3	*CCL5*	NM_002985.2
	*CXCL16*	NM_022059.2	*CCR6*	NM_031409.3;
				NM_004367.5
Cluster of differentiation	*CD24*	NM_013230.2	*CD28*	NM_006139.2
	*CD302*	NM_014880.4	*CD36*	NM_001001548.2
			*CD82*	NM_002231.3;
				NM_001024844.1
			*CD84*	NM_003874.2
			*CD96*	NM_198196.2 ;
				NM_005816.4
			*CD109*	NM_133493.3;
				NM_001159587.1;
				NM_001159588.1
			*SLAMF6*	NM_052931.3
			*SLAMF7*	NM_021181.3
HLA and related receptors	*FCAR*	NM_133279.2	*HLA-DOA*	NM_002119.3
	*FCGR2A*	NM_021642.3;	*HLA-E*	NM_005516.4
		NM_001136219.1		
			*LILRB1*	NM_006669.3;
				NM_001081637.1;
				NM_001081638.1;
				NM_001081639.1
			*LILRB3*	NM_001081450.1;
				NM_006864.2;
Transcription factors			*NFATC3*	NM_173164.1
			*NFATC2IP*	NM_032815.3
			*NFKBIB*	NM_001001716.1
			*TONSL (NFKBIL2)*	NM_013432.4
			*NKIRAS2*	NM_001001349.2;
				NM_017595.5;
				NM_001144927.1;
				NM_001144928.1;
				NM_001144929.1
Others	*NLRC3*	NM_178844.2	*PCM1*	NM_006197.3
			*DDX51*	NM_175066.3

**Table 5:** List of embryonic stem cell related genes in Ant-alus and Sens-alus

**Gene symbol**	**RefSeq accession**	**Gene name**
**Ant-alus**		
*ANGEL2*	NM_144567.3	Protein angel homolog 2
*CBX5*	NM_012117.2; NM_001127321.1; NM_001127322.1	Chromobox protein homolog 5
*CD24*	NM_013230.2	Signal transducer CD24
*CDC6*	NM_001254.3	Cell division control protein 6 homolog
*CDH1*	NM_004360.3	cadherin 1, type 1, E-cadherin (epithelial)
*CECR1*	NM_017424.2; NM_177405.1	Cat eye syndrome critical region protein 1
*DHFR*	NM_000791.3	dihydrofolate reductase
*DPPA4*	NM_018189.3	Developmental pluripotency-associated protein 4
*FAM108B1*	NM_001025780.1	Abhydrolase domain-containing protein FAM108B1
*GNPTAB*	NM_024312.3	N-acetylglucosamine-1-phosphotransferase subunit beta
*MICB*	NM_005931.3	MHC class I polypeptide-related sequence B
*NANOG*	NM_024865.2	Homeobox protein NANOG
*NUDT15*	NM_018283.1	Probable 7,8-dihydro-8-oxoguanine triphosphatase NUDT15
*PDK1*	NM_002610.3	3-phosphoinositide-dependent protein kinase 1
*PFAS*	NM_012393.	Phosphoribosylformylglycinamidine synthase
*PHF17*	NM_024900.3	Protein Jade-1
*RPS24*	NM_001142285.1	ribosomal protein S24
*RRM2*	NM_001034.3; NM_001165931.1	Ribonucleoside-diphosphate reductase subunit M2
*RRP15*	NM_016052.3;	RRP15-like protein
*SFRS1*	NM_006924.4; NM_001078166.1	Splicing factor, arginine/serine-rich 1
*TDGF1*	NM_003212.2	Teratocarcinoma-derived growth factor 1
*TERF1*	NM_003218.3; NM_017489.2	Telomeric repeat-binding factor 1
**Sens-alus**		
*ATP1A2*	NM_000702.3	Sodium/potassium-transporting ATPase subunit alpha-2
*CDT1*	NM_030928.3	DNA replication factor Cdt1
*DSG2*	NM_001943.3	Desmoglein-2
*LIN28*	NM_024674.4	Lin-28 homolog A
*MCM4*	NM_005914.2; NM_182746.1	DNA replication licensing factor MCM4
*NCAPH*	NM_015341.3	Condensin complex subunit 2
*NFYB*	NM_006166.3	Nuclear transcription factor Y subunit beta
*PAICS*	NM_006452.3; NM_001079524.1; NM_001079525.1	Phosphoribosylaminoimidazole carboxylase
*PIPOX*	NM_016518.2	Peroxisomal sarcosine oxidase
*PPM1B*	NM_177968.2	Protein phosphatase 1B
*PRDM14*	NM_024504.2	PR domain zinc finger protein 14
*PRKX*	NM_005044.3	Serine/threonine-protein kinase PRKX

One possibility for such a difference of Ant-alus and Sens-alus in pathway distributions is that certain genes underwent duplication events, after Alu retrotransposed into these genes, producing a number of paralogs of Alu-carrying genes associated to similar functions. As the primate-specific Alu incorporation events were fairly recent in evolution (~65 million years), these paralogs should still remain in the same protein subfamilies. To check this possibility, we checked the protein subfamilies among Ant-alus and Sens-alus. It was found that 96.2% of Ant-alus and 96.9% of Sens-alus have unique subfamilies (Additional file 1: Table S1, Additional file 1: Table S2), leaving 3.8% of Ant-alus and 3.1% of Sens-alus associated to the same protein subfamilies with others. This suggests that the gene duplication events accounted for a smaller fraction of pathway distributions than direct Alu retrotransposition. That said, gene duplication and Alu incorporation were both parts of evolution which jointly shaped the human genome and its biological functions. The functional annotation was thus based on the final set of Alu-carrying genes till this point in evolution.

### Significant suppression of Alu-tagged mRNAs by Alu perturbations

An extrachromosomal replication system was established to examine the perturbation of Alu-carrying genes in response to elevated Alu RNAs in the opposite direction. The null hypothesis here is that the Alu-carrying RNA duplex cannot trigger subsequent post-transcriptional regulation, manifesting a random fluctuation of expression levels. Transfected sense and antisense Alus were first checked to have expressed successfully, by the detection of chimeric RNA sequences expressed from the artificially constructed template sequence encompassing both vector and Alus.

**Figure 2:**
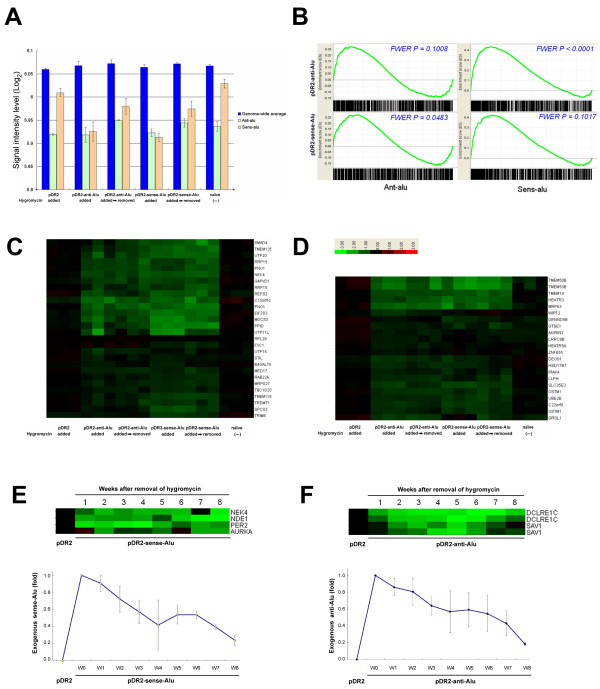
**Expression levels of Sens-alus and Ant-alus upon Alu perturbations. (A)** An overview plot of Sens-alus, Ant-alus and genome-wide RNA levels across 6 different treatment conditions. HEK293 cells were transfected with (1) empty pDR2 vectors (pDR2; Hygromycin added); (2) antisense Alus with Hygromycin selection (pDR2-anti-Alu; Hygromycin added; week 0); (3) the same as (2) with Hygromycin removed subsequently (pDR2-anti-Alu; Hygromycin added→removed; week 1); (4) sense Alus with Hygromycin selection (pDR2-sense-Alu; Hygromycin added; week 0); (5) the same as (4) with Hygromycin removed subsequently (pDR2-sense-Alu; Hygromycin added→removed, week 1); and (6) the original HEK 293 cells (no treatment). Vertical bars, means of expression levels per various gene sets from triplicate experiments. Error bars, standard deviations. **(B)** GSEA plots for the suppression effects of Ant-alus (upper left) and Sens-alus (upper right) in response to antisense Alu transfections, as well as sense Alu transfections (lower left and right respectively). NES, Normalized enrichment score. **(C)** The heatmap of a collection of Ant-alu genes which showed significant suppression individually upon sense Alu transfection, using cells transfected by empty vectors as controls (P< 0.0005, FDR < 0.0321). Green color indicated suppression. **(D)** The heatmap of a collection of Sens-alu genes which showed significant suppression upon antiense Alus transfection (P< 0.0005, FDR < 0.0518). **(E)** A heatmap representation of protein expressions of randomly selected Ant-alu genes, quantified at different time points by western blotting, up to the 8th week after the removal of Hygromycin. Protein levels of cells transfected by empty vectors (pDR2) were presented as baselines. Numbers in the time axis indicated weeks after Hygromycin removal. The time-course profile of extrachromosomal expression of sense Alu RNA was also presented. **(F)** Protein levels of randomly selected Sens-alu genes quantified at different time points. The time-course profile of exogeneous antisense Alu RNA was presented.

Genome-wide RNA expressions were measured in 6 different treatment conditions defined in the legend of Figure 2. Average levels of Ant-alu and Sens-alu were below the genome-wide average levels in all 6 conditions (Figure 2A). A Gene Set Enrichment Analysis (GSEA) was employed due to its capability of assessing the group behavior of a set of genes [26,27], a favorable feature for our examination of protein-coding mRNAs carrying Alus in opposite directions. As a group, Sens-alus were significantly suppressed in terms of family-wise error rate (FWER) (P < 0.0001), while Ant-alus were not significantly suppressed in response to antisense Alus transfection (P = 0.1008), using cells transfected by empty vectors as controls (Figure 2B, upper panels). In contrast, Ant-alus were significantly suppressed (P = 0.0483), while Sens-alus were not significantly suppressed in response to sense Alus transfection (P = 0.1017) (Figure 2B, lower panels). After the removal of selection antibiotics (Hygromycin), exogenous sense and antisense Alu RNAs gradually reduced and the two sets of mRNAs rebounded accordingly. At week 1, only Sens-alu were significantly different from week 0 (Ant-alu P = 0.2503, Sens-alu P = 0.0479).

In addition to the GSEA evaluation of group behaviors, we also performed analysis on individual probe sets. Ant-alus were selected if they manifested significant down regulation in response to sense Alu transfections (P< 0.0005, FDR < 0.0321). The fold change of RNA level was between 31.1% and 92.5%. Their expression levels across all 6 conditions were shown as a heatmap in Figure 2C. It showed that in addition to the suppression by sense Alus, the same set of genes can also be suppressed by antisense Alus. Sens-alus were selected if they manifested significant down regulation in response to antisense Alu transfetions (P< 0.0005, FDR < 0.0518). The fold change of RNA level was between 29.6% and 95.4%. Again, the heatmap showed that the same set of genes can also be suppressed by sense Alu (Figure 2D).

We also conducted a smaller-scale experiment for measuring the protein abundance of several randomly selected Sens-alus and Ant-alus, in response to the transfection of Alus in opposite directions, using western blotting. This time, the protein abundances were measured repeatedly once a week up to the 8th week after the selection antibiotics were removed (Figure 2E and 2F). The exogenous sense and antisense Alu RNAs gradually reduced to <25% at week 8 (compared with the maximum level at week 0), and protein suppression effects were observed during the period while the exogeneous Alu was still present.

## Discussion

### A regulatory network mediated by Alu RNA duplex

**Figure 3:**
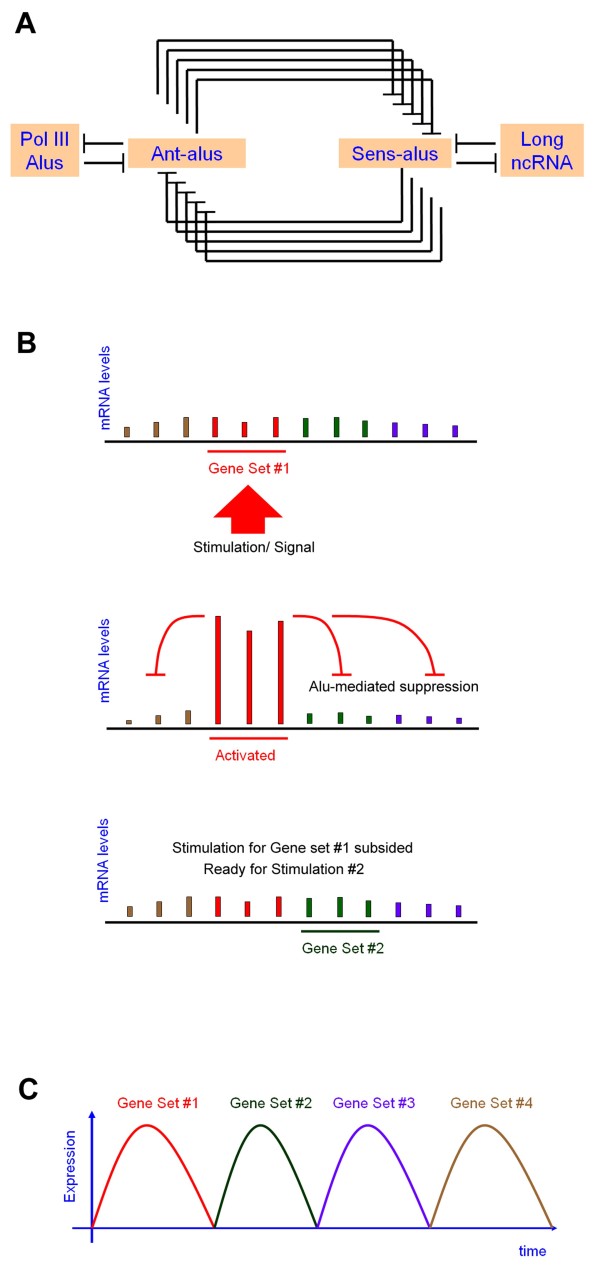
**Gene activations and suppressions mediated by Alu-carrying RNAs. (A)** A conceptual interaction network, where any two RNA species that may form a long (280~300 bp) Alu duplex were depicted by mutual inhibition signs. Central to the regulation network are Ant-alus and Sens-alus, which together represent 7.3% of total protein-coding genes. Their RNA levels may affect downstream protein levels. Pol III derived Alus may also form a binding with Ant-alus, enabling a mutual regulation. A few non-coding RNAs (ncRNAs) have also been reported elsewhere to bind with Sens-alus and then trigger STAU1-mediated mRNA decay. **(B)** The activation restriction model. A set of alu-carrying genes was activated and increased expression level in response to outside stimulation. The elevation of these genes increased the Alu element in the cytosol, which can suppress the activation of other Alu-carrying genes which are associated to other pathways. The suppression will continue until the original signal has subsided. Then a new activation can proceed. **(C)** Waves of genes are activated coordinately, with different set of genes activated in different time, due to Alu-mediated suppression.

Alus contribute a significant portion of the human genome. However, their cellular roles remain largely elusive. A better understanding of Alus’ roles can substantially enhance our overall knowledge on the human genome. We demonstrated that two species of mRNAs, harboring sense or antisense Alus respectively, could form a long RNA duplex longer than 290 bases. Also, the co-existence of sense and antisense RNAs in a cell can trigger group post-transcriptional regulation of two sets of Alu-carrying mRNAs. It is important to note that the intergenic, polymerase (pol) III-directed Alu RNA transcripts may also hybridize with Ant-alus due to similar thermodynamic base pairing. Further, long non-coding RNAs have been reported to hybridize with mRNAs with Alu elements [15]. Taken together, a static network of Alu-mediated interactions was conceptualized, comprising four Alu-carrying RNA species: Ant-alus, Sens-alus, Pol-III derived Alus, and long non-coding Alu-carrying RNAs (Figure 3A). At the center stage are protein-coding Ant-alus and Sens-alus. An altered expression of any species may tilt the balance of the entire system, thereby changing cellular states.

What could be the major driving forces for the dynamics of the network? Environmental stimuli such as stress may be one answer. It was reported that Pol III derived Alu transcripts, usually dormant in normal cell conditions, were elevated by stress such as viral infection [9,17,28]. Pol III Alu may be perturbed together with all the other species upon stress response, although the physiological level of perturbation of the four RNA species remained elusive.

Data from the in-vitro system showed that the Sens-alus and Ant-alus were suppressed significantly by transfected Alu counterparts. The transfected sense Alu could represent over-expressed Sens-alus, or the Pol-III derived Alus, as both of them have similar Alu elements in the sense direction to suppress its counterparts. Likewise, the transfected antisense Alu could represent over-expressed Ant-alus, or long non-coding transcripts with antisense Alu elements.

The strong binding of genes with opposite Alu directions was predicted by the RNA folding algorithm. The empirical evidence of the binding was still lacking. We have been planning an experiment based on the idea of using multiple Alu-carrying genes as baits. A binding column will be used to capture the baits. Those RNA bind to the baits can also be captured and then analyzed. This however remained to be our future work.

The network of the Alu-carrying RNAs may underlie the stability and transitions of human cellular states such as neurological or immunological response, as was suggested by the functional annotations of protein-coding Alu-carrying mRNAs. First, the mutual suppression effect may offer barriers among cell lineages. Random fluctuations of Alu-carrying genes may be restricted by the network. Second, upon the invasion of pathogens, the immune system must respond quickly to turn the immature immune cells into mature states by coordinated activations of genes, many of which are Sens-Alus (Table 4).

### Activation restriction for gene expression cascades

Previous work by Vidal and colleagues showed that the B1-repeat elements are necessary rather than sufficient criteria for the co-expression of genes, implying that some, but not all, B1-containing genes are activated concurrently [23]. Developmental processes, neurological and immunological functions have been known to comprise many signal transduction events. We continued to reason that, when a signal transmits to a set of Alu-containing genes, they may be activated by way of elevation of their expression levels. The Alu-mediated suppressing effect offers a built-in inhibitory mechanism toward other Alu-tagged RNA species despite the presence of their individual activation signals from the noisy environment. The net effect is a restricted activation of one or few signal transduction, while other temporarily unwanted signals are filtered away. This situation will persist till the formal signaling effect has subsided. Then, another signaling can come through, resulting in a sequence of gene expressions. A full scale of disordered responses is thus prevented, and the activation of Alu-containing genes can proceed in a coordinated fashion, one state after another (Figure 3B and 3C).

One key question about the mutual regulation of Sens-alus and Ant-alus is the responsible molecular mechanisms. Is it through the *Dicer1*-created siRNA mechanism, the STAU1-mediated RNA degradation, or both? Our data suggested that *Dicer1* may play a bigger role. An interesting observation from our experiments is that Alu-carrying genes can be suppressed by transfected Alus in the same direction, although not to the level of statistical significance. These may be explained by the potent source of siRNA offered by the duplex of transfected sense Alu and Ant-alu, upon the cleavage of *Dicer1*, which may suppress both Sens-alus and Ant-alus depending on the guide strand directions [18]. Sens-alus were thus suppressed by the RNA-induced silencing complex using siRNAs in the antisense strand as the guide strand. Interestingly, *Dicer1* is also down regulated in chemically stressed cells [3]. Recent data also showed the intimate trade-off between *Dicer1* and Alu abundance [10].

## Conclusions

In summary, we proposed a complex regulation network mediated by the Alu “tags” in four species of RNAs and offered initial evidence. The Alu-mediated suppression effect may restrict the activation of genes with other “tags”, thereby stabilizing state transitions observed along cellular lineages or in response to outside stimuli. Additionally, different ratios of Sens-alus and Ant-alus may be observed in different types of human cells, with two extreme examples of Ant-alus or Sens-alus as the predominant constituents of expressed genes. The former state may be related to neurological functions, while the later may be related to immunological functions.

## Methods

### Cell-based assay

An in-vitro extrachromosomal replication system was established to examine the postulated regulation effects on genes carrying the Alu elements. Sense and antisense Alus were cloned from cDNAs, which were reversely transcribed from mRNAs of *PCM1* (nt 7636 to nt 8082; [RefSeq:NM_006197.3]) and *PER2* (nt 5221 to nt 5781; [RefSeq:NM_022817.2]), respectively. The clones were then inserted into pDR2 vectors (Clontech, Mountain View, CA) downstream of the Rous sarcoma virus long terminal repeat (LTR) promoter. This plasmid vector contains Epstein-Barr virus *OriP*, a gene for hygromycin B selection, and an ampicillin resistance gene. These two plasmids, and a pDR2 vector carrying no additional DNA (the empty vector), were transfected to human embryonic kidney cells constitutively expressing Epstein-Barr virus nuclear antigen-1 (EBNA-1) protein from Epstein-Barr virus (Hek293EBNA cells; Invitrogen, Carlsbad, CA). The three cell lines were maintained in Dulbecco’s modified Eagle’s medium containing 10% fetal bovine serum and 250 ug of G418 per ml. Hygromycin (0.6 mg/ml) was added to the cell culture medium for the selection of stable transformants. After the transfection, the RNA extracts were submitted for RT-PCR, cloning and sequencing to check whether the expressed RNA encompasses both the vector part and the inserted sequence, an evidence that the transfected sequence has successfully expressed in our system. Real-time PCR was also performed to monitor the expression levels of the exogenous transcripts weekly after removal of hygromycin from the culture medium. Upon removal of hygromycin, the extrachromosomal replicating plasmids were gradually lost, allowing for reversion to the un-transfected status.

The mRNA of Sens-alus and Ant-alus, in response to the transfection, were measured by gene expression microarray. Affymetrix Human PrimeView™ arrays were used (Affymetrix, Santa Clara, CA). An in vitro transcription (IVT) with biotinylated ribonucleotide analog were then performed to generate biotin-labeled amplified RNA (aRNA), using GeneChip 3′IVT Express kit (Affymetrix, Santa Clara, CA). The aRNAs were then purified by magnetic beads and fragmented for the subsequent hybridization according to the manufacturer’s protocol. Fluorescent signal was scanned by GeneChip Scanner 3000 7G (Affymetrix, Santa Clara, CA) to produce digital images and then converted and summarized to intensity readings per probe sets (total n= 49395). The protein expression levels of several Alu-containing genes were assayed by western blotting. The microarray raw and normalized data can be found on the NCBI GEO repository by the accession number GSE39822.

### Bioinformatics

RNA secondary structures of full length mRNA of *PER2* and *PCM1* were predicted using the standalone RNA-cofold software offered by the Vienna RNA group [29,30].

The Alu elements in *PCM1* (nt 7691 to nt 8008; [RefSeq:NM_006197.3]) and *JAK3* (nt 4299 to nt 4614; [RefSeq:NM_000215.3]) were used as query sequences to search against the entire NCBI-RefSeq database [25] for antisense hits using the command-line Yass alignment software [31]. Standard parameters were used, and hits must have e-values smaller than 10^-20^ and length longer than 290 bases. This parameter setting allowed non-perfect matches. The coding regions annotated by NCBI-Refseq were also used to discern whether the antisense hits were located in 5′UTR, 3′UTR or the coding regions.

Protein-coding genes were sieved from the Alu-carrying transcripts using the PANTHER (Protein ANalysis THrough Evolutionary Relationships) Classification System version 7.2 on the official bioinformatic site [32]. PANTHER is a sequence based, phylogenic-tree supported system with protein functions annotated by human experts, ensuring a high quality of annotation. Sens-alus were defined by genes with Alus only in the sense direction while Ant-alus were defined by genes with Alus only in the antisense direction. The subfamilies of Sens-alus and Ant-alus were assigned by PANTHER. Functional annotations of over- or under-representation of Ant-alus and Sens-alus amongst various pathways and biological functions were also performed by PANTHER. Gene symbols of 689 and 771 genes were submitted to the website, and the functional annotations of pathways and biological processes of the two lists of genes were calculated concurrently by the system. A total of 165 pathways and 212 biological processes were checked individually to see the level of over- and under-representation of the two lists of genes. The P values were derived using the binormial distribution tests. False discovery rates (FDR) were also calculated to accompany the P values, addressing issues of multiple comparisons. The downloadable results were in the format similar to Tables 2 and 3.

The expression levels across all 18 microarrays (for 6 conditions, each with three biological replicates) were normalized using the RMAExpress (version 1.0.5), implementing the Robust multiarray analysis (RMA) algorithm [33-35]. Gene expression levels per probe set were compared across groups using unpaired two sample t-test assuming unequal variance. False discovery rates (FDR) were used to assess significance in the scenario of multiple comparisons. All P-values were two-tailed.

Perturbation of gene expression levels were evaluated by the stand-alone GSEA software v2.07 offered by the Broad Institute [26,27]. The goal was to analyze the global perturbations of set of genes of interest, by sense and antisense Alu transfections, in comparison with the control samples of Hek293 cells transfected by empty vectors. GSEA examines whether particular sets of genes (in our case, Ant-alus and Sens-alus) tend to be the leading perturbed genes amongst all genes. When multiple probe-sets are associated to a gene, the median of all probe-set measurements were used to represent the gene. The perturbation was quantified by the difference of gene level between two treatment conditions (i.e. classes). Family-wise error rate (FWER) P-values were derived from an empirical distribution upon 10000 permutations of the class labels to address multiple comparison issues.

The RNA and protein expressions were visualized as heatmaps using Cluster 3.0 [36,37] and TreeView version 1.1.6r2 [38]. In the heatmap presentation, the expression levels were subtracted by baseline values which were the average measurements on naïve cells and cells with empty vectors.

## Abbreviations

Ant-Alu: Antisense Alu-carrying messenger RNA; Sens-Alu: Sense Alu-carrying messenger RNA; L1: Long interspersed nucleotide element 1; UTR: Untranslated regions; GSEA: Gene Set Enrichment Analysis; FDR: False discovery rate; FWER: Family-wise error rate; RMA: Robust multiarray analysis.

## Competing interests

The authors declare that they have no competing interests.

## Authors’ contributions

Both KHL and CTY conceived the project and proposed the hypotheses. KHL carried out the bioinformatics analysis. CTY produced the extrachromosomal replication system and carried out the experiments. Both KHL and CTY drafted the manuscript and drew the conclusions together. Both authors have read and approved the final manuscript.

## Supplementary Material

Additional file 1: Table S1 The complet list of Ant-alus. **Table S2.** The complet list of Sens-alus. **Table S3.** The complet list of protein coding genes which have Alu elements in both sense and antisense directions. Click here for file
